# Central nervous system metastasis secondary to colorectal cancer: a retrospective cohort study of 20 cases

**DOI:** 10.3332/ecancer.2016.705

**Published:** 2016-12-21

**Authors:** Sebastián Mondaca, Valentina Hornig, Pablo Munoz-Schuffenegger, Francisco Acevedo, Marcelo Garrido, Bruno Nervi

**Affiliations:** 1 Departamento de Hematología-Oncología, Oncología Médica, Facultad de Medicina, Pontificia Universidad Católica de Chile, Diagonal Paraguay 319, Santiago, Chile 8330032; 2 Departamento de Hematología-Oncología, Radioterapia, Facultad de Medicina, Pontificia Universidad Católica de Chile, Diagonal Paraguay 319, Santiago, Chile 8330032; 3 Department of Radiation Oncology, University of Toronto, Toronto, ON, Canada; 4 Department of Radiation Oncology, Sunnybrook Health Sciences Centre, Odette Cancer Centre, Toronto, ON, Canada

**Keywords:** Colorectal neoplasia, cerebral metastasis, radiosurgery, neurosurgery

## Abstract

**Introduction:**

Involvement of the central nervous system (CNS) secondary to colorectal cancer is infrequent and associated with a poor prognosis. Its treatment is extrapolated from metastases of other origins as the information available on this scenario is limited. The goal of this study is to assess the clinical characteristics of a series of patients and determine the results in terms of progression-free survival (PFS) and global survival.

**Method:**

The records of patients with CNS metastasis of colorectal origin who were treated in this facility between the years 2001 and 2016 were reviewed retrospectively.

**Results:**

20 patients with CNS lesions of this origin were identified. Of these, 45% were male and 55% were female (average age 65.5 years). The histology corresponded to tubular adenocarcinoma in 95% of cases. Around 85% of the patients showed a neurological deficit, and their recursive partitioning analysis (RPA) classifications were 1 in 20%, 2 in 55%, and 3 in 25% of the cases studied. The treatments provided were: holocerebral radiotherapy (45%), stereotactic radiosurgery (25%), surgery followed by holocerebral radiotherapy (25%), and exclusively palliative care (5%). The PFS was 2.6 months from treatment of the CNS lesion, while the median survival was 3.8 months. The survival times for patients receiving different treatments were as follows: surgery plus holocerebral radiotherapy 16.2 months, stereotactic radiotherapy 12 months, and holocerebral radiotherapy 2.4 months (p = 0.003).

**Conclusion:**

The prognosis for patients with metastasis of colorectal origin is poor. The patients treated with surgery or stereotactic radiotherapy can have a greater survival.

## Introduction

Colorectal cancer (CRC) is the third most common cancer and the fourth in mortality at the global level [1], and it is a public health issue for both developed countries and developing countries. Around 20% of patients show metastatic disease at the time of diagnosis and up to a third of patients may develop it, particularly in the liver [2]. The central nervous system (CNS) is an infrequent site of involvement, representing 1–4% of cases in different studies [3, 4], however, epidemiological data show an increasing trend in its occurrence [5, 6]. This can be explained further because of the success that has been achieved in managing extracranial metastatic illness. A recent systematic review reports that CRC is the principal cause of CNS metastasis of gastrointestinal origin representing 79% of the total [7]. In patients with CRC, the presence of CNS involvement tends to be delayed and is associated with a poor prognosis with a median survival of about four months [8] which compares unfavorably with that of patients with CNS metastasis of other origin [9]. In the past few years, significant advances have been made in the field of neuroanesthesia, neurosurgical instruments, and techniques for stereotactic radiosurgery which have shown benefits in the treatment of patients with cerebral metastases overall [10, 11]. However, it has not been demonstrated that these treatments are effective in patients with CNS lesions of colorectal origin. In addition, the published studies are mainly of North American and Asian populations, and thus the possibility of extrapolating this data to other non-represented populations is limited.

The goal of this study is to retrospectively assess the clinical characteristics of a series of Latin American patients with CNS metastasis of colorectal origin and determine the results in terms of PFS and overall survival.

## Method

A retrospective analysis of the database of patients treated with a diagnosis of CRC between the years 2001 and 2016 in the cancer centre of the Pontifica Universidad Católica de Chile was conducted. The diagnosis of CRC was reached through histological confirmation, and the clinical files of the patients were reviewed in order to extract the relevant clinical data. We used the American Joint Committee of Cancer (AJCC) TNM seventh edition classification for staging purposes. The diagnosis of CNS involvement was made using computed tomography (CT) with iodated contrast or magnetic resonance imaging (MRI) of the brain with gadolinium. The information on mortality was extracted from the Chilean Civil Registry Service database. The characteristics of the population were expressed as a mean for continual variables and as percentages for categorical variables. The statistical analysis was done using Kaplan-Meier curves with a log-rank test for the survival analysis. The programmes SPSS Version 21 and GraphPad Prism 7.0 were used to analyse the data.

Dispensing with consent was requested for this study and was approved by the institutional ethics committee of the Pontificia Universidad Católica de Chile.

## Results

### Patients

Of a total of 1154 patients with CRC who were treated during the time period of the study, we identified 20 patients with CNS metastasis of colorectal origin which corresponds to 1.7% of the total. Of these, 45% were male and 55% were female. The average age was 65.5 years (range between 42 and 84). The histological diagnosis corresponded to tubular adenocarcinoma in 95% of the patients, and 20% had CNS involvement at their initial consultation. The location of the primary tumour was in the colon in 70% of the cases (Table 1). The stage according to TNM at the time of diagnosis of the patients was Stage II: 10%, Stage III: 35%, and Stage IV: 55% of which 80% of the patients received chemotherapy prior to the diagnosis of metastatic involvement of the central nervous system, and a 50% of them also received biological medications such as bevacizumab, cetuximab, or panitumumab.

A total of 65% of the patients showed a single metastasis at the time of diagnosis of intracranial illness. The location of the involvement of the CNS was supratentorial in 65% of the patients, infratentorial in 25%, and the remaining 10% had involvement in both locations.

Among all 85% of the patients showed a neurological deficit, and their RPA classification [12] was 1 in 15%, 2 in 65%, and 3 in 25% of the patients (Table 2). Seven patients in the group were tested for the KRAS mutation which was present in one of them.

### Treatment and progression

The average follow-up was 7.6 months. In this series, only 5% of the patients were treated exclusively with palliative care associated with the use of systemic steroids. A total of 65% were treated with holocerebral RT with a dose between 19 and 39 Gy which includes the suspension of treatment because of complications or clinical deterioration. Among all 25% were treated with stereotactic radiosurgery and 25% with surgery followed by holocerebral RT. Of the five patients treated with surgery, a complete resection (R0 resection) was achieved in four, while in one case residual microscopic illness was evident in the biopsy (R1 resection).

**Figure 1.**
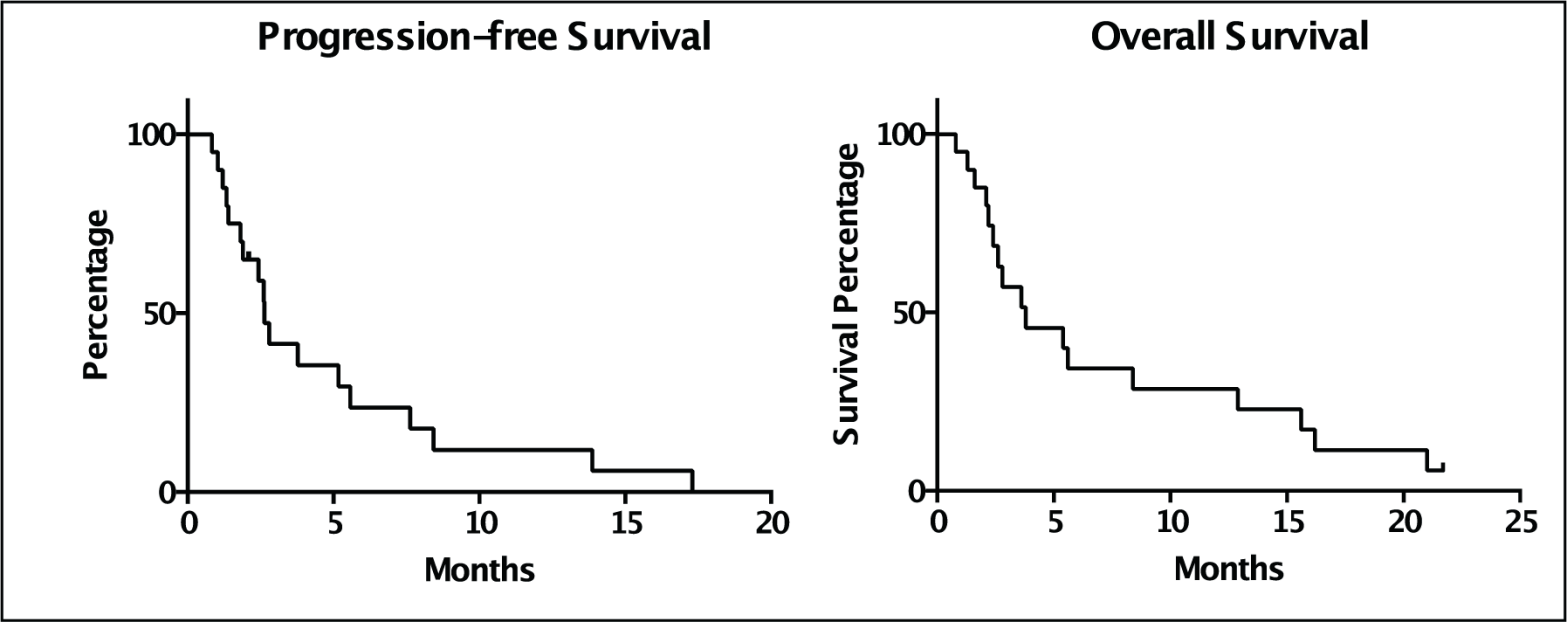
Kaplan Meier Curves for the assessment of progression-free survival and overall survival.

**Figure 2.**
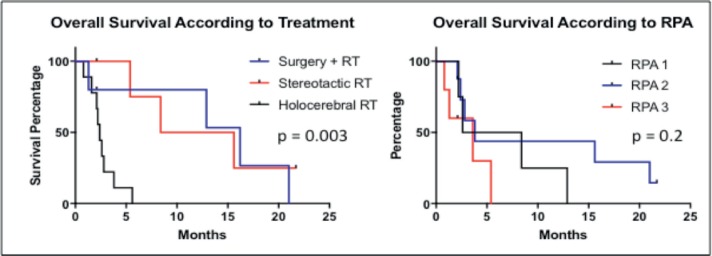
Kaplan Meier Curves for the assessment of progression-free survival and overall survival according to treatment and RPA.

The PFS was 2.6 months from treatment of the CNS lesion, while the overall survival (OS) was 3.8 months. Survival at one year was 20%, and at two years was 5% (Figure 1). The subgroups treated with stereotactic radiosurgery or with surgery plus holocerebral RT had a survival of 12 and 16.2 months respectively. The subgroup treated only with holocerebral RT had a median survival of 2.4 months p = 0.003 (Figure 2), while the patient treated with palliative care had a survival of 3.6 months. The OS according to RPA was 5.5 months for one, 3.8 months for two, and 3.6 months for three, p = 0.2 (Figure 2). Of the patients, 35% received systemic chemotherapy following treatment of the intracranial illness.

## Discussion

Our retrospective study of 20 patients with CNS involvement of colorectal origin confirms a poor prognosis for this group with a median survival of 3.8 months. One patient had a survival of more than two years which supports the use of aggressive treatment based on surgery or stereotactic radiosurgery. Most modern studies showed a similar survival compared to ours of about five months [7, 13, 14], although other studies have reported a better survival of nearly ten months [15]. The survival of patients treated with stereotactic radiosurgery or surgery followed by holocerebral RT was markedly greater than that of patients treated only with holocerebral RT. This finding can be explained by the selection bias associated with the fact that patients with factors indicating a poor prognosis were treated with holocerebral RT. Regarding this point, there are studies in which multivariant analysis validates the type of treatment as a factor in the prognosis [15, 16], while others have indicated the opposite [17, 18]. RPA is a validated prognostic classification which is often used in clinical practice which considers the factors related to the general condition of the patient and the control of systemic extracranial disease [12, 19]. Our study did not show significant variation in survival according to RPA classification. Another issue that should be noted is that of the high level of exclusive infratentorial involvement which was observed in a quarter of the patients. This has been described in other studies too where they have observed the presence of infratentorial lesions in between 22 and 55% of the patients with CRC and CNS metastasis [20]. A relevant limitation of our study is the relatively small number of patients, which makes it impossible to perform a multivariant analysis of prognostic factors. In addition, the data was collected retrospectively, so there was not a strict protocol for the diagnosis or treatment of these patients. For this reason there is a significant selection bias for the various treatments which prevents us from reaching definitive conclusions on their effectiveness. Among the strengths of this study is the fact that the treatment was provided in a single academic centre which has a tumour registry for the identification of patients. To our knowledge, this is the first study on Latin American patients with CNS involvement of colorectal origin. This has a relevance by becoming the inclusion data which represents different populations.

The prognosis for patients with metastatic CRC has significantly improved in the past few years, however, there is still no clinically relevant molecular classification. In this sense we believe that the model of patients with CNS metastasis could be interesting for the development of biomarkers and understanding the mechanisms of metastasis, as has been suggested by other research groups [21].

## Conclusion

In conclusion, our study supports the poor prognosis of the patients with CNS involvement of colorectal origin and suggests that patients treated in an aggressive manner with surgery followed by holocerebral RT or stereotactic radiosurgery can have a longer survival even exceeding two years.

## Figures and Tables

**Table 1.** Basal patient characteristics.

	Patients (N = 20)
Age (years)	65.5
Sex (%) Male Female	45 55
Location (%) Colon Rectum	70 30
Histology (%) Adenocarcinoma Mucinous Other	95 0 5
AJCC Stage (%) II III IV	10 35 55
Tumour grade (%) 1 2 3	10 80 10
Prior Chemo (%) Yes No	80 20
Prior biological treatment (%) Yes No	50 50

Chemo: Chemotherapy

**Table 2.** CNS involvement characteristics.

	Patients (N = 20)
Number of lesions (%) Single Multiple	65 35
Location (%) Supratentorial Infratentorial Both	65 25 10
Lesion size (%) More than 3 cm Less than or equal to 3 cm	N = 18 28.6 61.9
Time profile (%) Synchronic Metachronic	20 80
Neurological deficit (%) Yes No	85 15
RPA Classification (%) 1 2 3	20 55 25
Treatment (%) Holocerebral RT Stereotactic RT Surgery and holocerebral RT Palliative care	45 25 25 5
Posttreatment chemo (%) Yes No	35 65

CNS: Central nervous system

RPA: Recursive partitioning analysis

RT: Radiotherapy

Chemo: Chemotherapy
